# Field Displacement of Aflatoxigenic *Aspergillus flavus* Strains Through Repeated Biological Control Applications

**DOI:** 10.3389/fmicb.2019.01788

**Published:** 2019-08-07

**Authors:** Mark A. Weaver, Hamed K. Abbas

**Affiliations:** Biological Control of Pests Research Unit, United States Department of Agriculture, Agricultural Research Service, Stoneville, MS, United States

**Keywords:** aflatoxin prevention, biopesticide, mycotoxin, biological control, atoxigenic

## Abstract

A popular pre-harvest strategy to mitigate aflatoxin contamination of corn involves field application of non-aflatoxigenic strains of *Aspergillus flavus*. The basis of this biological control may involve multiple factors, but competitive displacement of aflatoxigenic strains by the biocontrol strains is a likely mechanism. Three biocontrol strains (NRRL 21882, 18543, and 30797) were applied annually, over a 4-year period, to the same 3.2-ha commercial corn field in the Mississippi Delta, where we monitored their post-release establishment, spread, and persistence. Within 2 months of the first biocontrol application, the percentage of soil-inhabiting aflatoxigenic *A. flavus* strains in some plots was reduced from 48 to 9% of the population. The frequency of aflatoxigenic *A. flavus* strains was also significantly reduced in the rest of field. After 4 years, neighboring plots that had never received a biocontrol treatment, and distanced from our treatment plots by at least 20 meters, had less than 20% aflatoxigenic isolates. This significant halo effect might be attributed to movement of soil through tillage operations, but the aflatoxigenicity shift could be detected in the untreated plots within 2 months of the initial applications, at a time when there was no tillage. The *A. flavus* populations that colonized the grain were also monitored and found to be less than 15% toxigenic in the fourth year for all treatments. Over all treatments and years, less than 2 ppb of aflatoxin was detected, which could be a consequence of the field-wide shift of the inherent *A. flavus* population to predominately non-aflatoxigenic strains. This study supports the efficacy of using non-aflatoxigenic *A. flavus* strains as pre-harvest biocontrol, and shows that most of its effectiveness occurs with the first application.

## Introduction

*Aspergillus flavus* is a common soil saprophyte and an opportunistic plant pathogen. Many agriculturally important crops can be infected by *A. flavus*. The resulting infections rarely cause appreciable yield loss, but can be important due to the resulting contamination by *A. flavus* mycotoxins. Several mycotoxins can be produced by *A. flavus* including aflatoxin (AF) and cyclopiazonic acid (CPA). Aflatoxin AF, a Group 1 carcinogen (IARC, 1993), is produced in several forms by various fungal species, and aflatoxin B_1_ is considered an especially potent liver carcinogen. Aflatoxin contamination of food and feed is especially problematic in parts of the world where the lack of testing and proper storage conditions lead to periodic aflatoxicosis, reduced growth in infants and children, and elevated risk of cancers (Wild et al., 2015). Europe and North America have enacted strict controls on the allowable levels of AF in foods and feeds, and the agricultural industry cooperates in monitoring AF throughout the food chain. Globally, the commodity that is most affected by AF is corn, resulting in tens to hundreds of millions of dollars in lost value annually. The level of economic loss varies greatly from year to year, but one model indicated a loss of up to US$ 1.06 billion in 2012, when weather conditions in the upper Midwest were particularly conducive to *A. flavus* infection and subsequent AF contamination, with corn prices at or near record highs (Mitchell et al., 2016). While there is comparatively less corn grown in the Southern U.S., it is contaminated by AF more frequently and at higher concentrations (Jones et al., 1981; Diener et al., 1987; Payne, 1992; Mitchell et al., 2016).

With these strong economic incentives, corn producers endeavor to reduce AF contamination. Because *A. flavus* is an opportunistic pathogen, efforts to promote host plant health and minimize plant stress may be helpful, but are often insufficient (Bruns, 2003; Wiatrak et al., 2005). AF contamination linked to insect activity, especially by the European Corn Borer (*Ostrinia nubilalis*) and Fall Armyworm (*Spodoptera frugiperda*), led to the expectation that transgenic insect control would reduce AF contamination (Dowd, 2001; Abbas et al., 2013). *Bt*-corn, developed to prevent host damage from insect predation, has been shown to be effective in the management of other mycotoxins, *e.g.,* fumonisin (Munkvold et al., 1999), but success with AF management by *Bt* insect control has been equivocal (Bowen et al., 2014; Weaver et al., 2017a,b). The most effective AF-reducing strategy is the pre-harvest application of *A. flavus* isolates that are naturally incapable of aflatoxin production. This strategy has been commercialized in the U.S. *via A. flavus* strain NRRL 21882 (AflaGuard GR, Syngenta Crop Protection, Greensboro, NC) and NRRL 18543 (AF36, Arizona Cotton Research and Protection Council), whose effectiveness has been repeatedly validated in U.S. field trials (Brown et al., 1991; Cotty, 1994; Bock et al., 2004; Dorner, 2010; Weaver et al., 2015), Europe (Mauro et al., 2018), and Africa (Atehnkeng et al., 2008; Bandyopadhyay et al., 2016).

While overall efficacy of this particular biocontrol approach for aflatoxin is well supported, questions remain regarding the multi-year dynamics of the indigenous *A. flavus* population in the context of biocontrol applications. We describe here an experiment involving 4 years of annual biocontrol applications, in a highly productive corn monoculture, to monitor *A. flavus* populations in the soil and in host kernel tissue. This experiment tested the hypotheses that applied biocontrol strains (1) displace the indigenous toxigenic strains, (2) persist beyond year of application; and (3) shift the indigenous *A. flavus* populations in a non-treated plot that is situated at a distance beyond the application site.

## Materials and Methods

### Fungal Strains and Inoculum Preparation

*Aspergillus flavus* strains NRRL 21882, 18543 and 30797 are non-aflatoxigenic biocontrol strains that have been well characterized (Abbas et al., 2011) and sequenced (Weaver et al., 2017a,b). These strains were produced on wheat that had been moistened and autoclaved on two consecutive days, as described previously (Abbas et al., 2006). Briefly, bags containing 1,600 g of sterilized wheat were inoculated with each biocontrol strain and incubated, mixing twice per day, for 4 days before drying in a forced air drier set at 30°C. Autoclaved, but not inoculated, wheat was also dried for use as a control treatment in field applications.

### Study Site

The field experiment was conducted from 2012 through 2015 in Washington County, Mississippi (Lat: 33.43°N; Lon: 90.94°W) on a 4-ha commercial corn field in continuous corn production. The soil type was a Bosket, very fine sandy loam without irrigation. The cultural and weather conditions are described in Table 1. Field preparation included fall deep tillage and raised beds to promote drainage, followed by tillage and reshaping of the beds in the spring.

**Table 1:** Growing conditions.

			Avg. Max/Min air temperature (°C)^*^		Precipitation (mm)^*^	
Year	Planting date	Hybrid planted	April–May	June–July	April–May	June–July
2012	3/20/2012	Rev 28R10	28/16	33/22	158	278
2013	4/10/2013	P16–15	24/13	31/20	312	141
2014	3/21/2014	DK66–97	25/14	31/21	407	268
2015	3/31/2015	DK62–05	27/16	34/23	317	136
Historical average			26/14	32/21	266	185

* Meteorological conditions recorded by Mississippi State University: http://deltaweather.extension.msstate.edu

**Figure 1:**
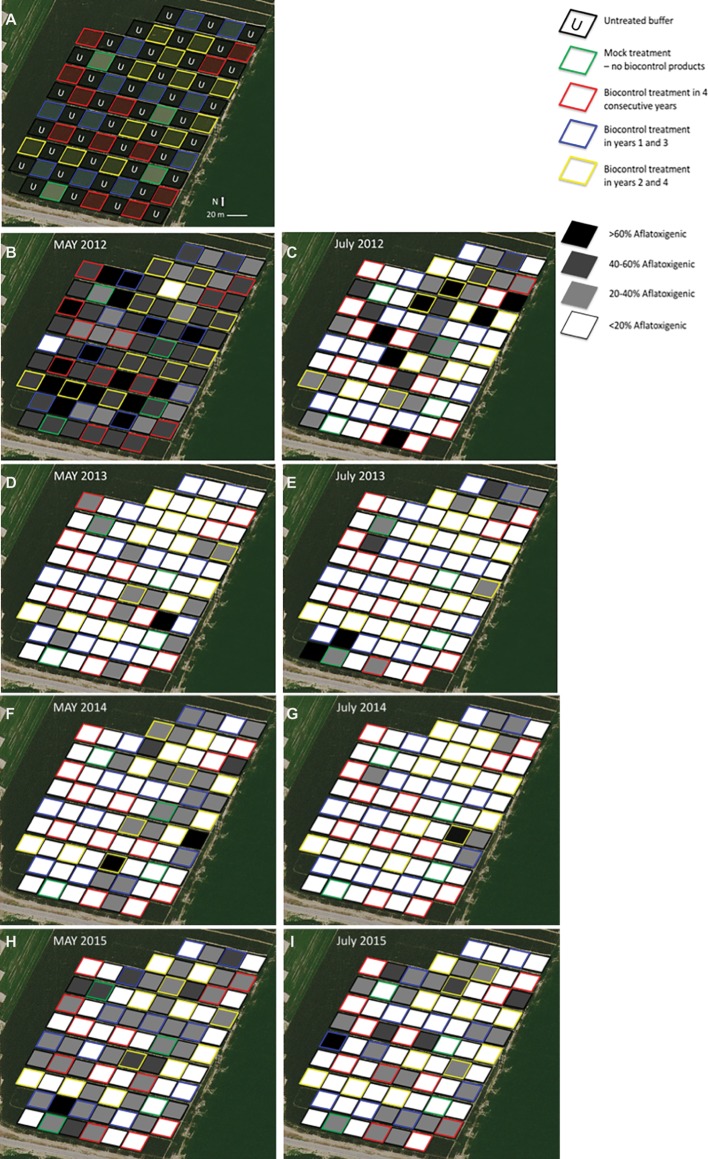
Spatial arrangement of treatment plots within the commercial corn field and percentage of the *A. flavus* population that was aflatoxigenic within each plot at a particular time. The same plot borders were maintained throughout the 4 years of the experiment. Plot borders are color coded to indicate the treatments received and shaded to indicate the percent of *A. flavus* isolates from a plot that were aflatoxigenic. **(A)** clearly presents the treatments and experimental layout. **(B–I)** present the *A. flavus* population dynamics.

Planting involved twin rows on raised beds with 97-cm spacing. Weed and insect pressures were closely managed to promote crop health. Plots were established by measuring 400-m^2^ (20 rows × 20 m length) areas in April of 2012, geo-referencing the position of the plots and marking plot borders. The same plot borders were maintained throughout the 4-year study. Surface soil samples (0–5 cm, each a composite of five subsamples, approximately 400 g in total, near the center of every plot) were collected in May of each year from every plot, immediately before biocontrol applications, and once more in July, coinciding with kernel development and potential *A. flavus* infection. Biocontrol applications were made with hand-held fertilizer spreaders (Scotts 71,133) at a rate of 22 kg ha^−1^, consistent with label guidelines for AflaGuard GR when corn was at the five leaf-collar stage (V5). There were four replications of each treatment in a randomized complete block. To minimize interference between treatments, a 400-m^2^ untreated buffer plot was included between all treatment plots. Treatments are described in Table 2, and the field layout is presented in Figure 1A. Grain was collected, by combine harvest, from the center two rows of each plot.

**Table 2:** Description of treatments.

	Applied inoculum			
Treatment	2012	2013	2014	2015
1. Control	Mock	Mock	Mock	Mock
2. Strain 21,182	21,182	21,182	21,182	21,182
3. Strain 30,797	30,797	30,797	30,797	30,797
4. Strain 18,543	18,543	18,543	18,543	18,543
5. Alternating 21,182	21,182	Mock	21,182	Mock
6. Alternating 21,182	Mock	21,182	Mock	21,182
7. Alternating 30,797	30,797	Mock	30,797	Mock
8. Alternating 30,797	Mock	30,797	Mock	30,797
9. Alternating 18,543	18,543	Mock	18,543	Mock
10. Alternating 18,543	Mock	18,543	Mock	18,543

### Sample Processing and Analysis

Soil samples were mixed with 0.1% Triton water and plated onto modified dichloran rose Bengal (MDRB) semi-selective medium to enumerate the *A. flavus* population (Horn and Dorner, 1998). Individual colonies were transferred to potato dextrose agar supplemented with β-cyclodextrin as a fluorescence enhancer and scored as presumptive aflatoxigenic or non-aflatoxigenic based upon production of a characteristic yellow pigment and a blue fluorescence. This methodology of Abbas et al. (2004) was used, except the scoring was done in 24-well culture dishes so that 22 isolates could be grown and scored alongside one known toxigenic strain and one known non-aflatoxigenic reference strain. This method was validated for 1,698 isolates by HPLC measurement of AF production, with the visual screen producing 89.93% accurate determination of toxigenicity.

A subsample of corn from each plot, weighing approximately 2 kg, was ground until at least 70% of the sample passed a 20-mesh screen, as per industry protocol (Texas State Chemist). A 50-g portion was extracted with 70% methanol for detection and quantification of AF, CPA, and fumonisin (Weaver et al., 2017a,b). An additional 20-g portion was mixed with 0.1% Triton water to isolate and characterize the *A. flavus* population using the same methods as the soil samples.

The colonization of the soil and grain by the non-aflatoxigenic strains was evaluated by analysis of variance (JMP 12.2, SAS Institute) at *α* = 0.05. For each year, the percent of non-aflatoxigenic isolates in the soil was compared between the May and July sample dates to determine the effect of biocontrol product applications in that season. No significant differences between the three biocontrol strains (21,882, 18,543, and 30,797) were detected in any year (2012, *p* = 0.08; 2013, *p* = 0.34; 2014, *p* = 0.34; 2015, *p* = 0.75), so they were grouped together in the analysis. Similarly, there were no significant differences between the three biocontrol strains applied in alternating years, so these treatments were also considered together. Analysis of variance and Tukey’s honestly significant difference test at *α* = 0.05 were used to evaluate treatment effects on the *A. flavus* isolates that colonized grain, as measured at harvest.

## Results and Discussion

### Mycotoxin Observations

Aflatoxin contamination is most common during periods of severe stress to the plant host, such as periods of high heat and drought, but is periodically a problem even in comparatively moist, highly productive sites (Abbas et al., 2012). This study took place during a period of historically high corn prices, leading to increased corn planting in the Mississippi Delta and supporting agronomic practices to maximize plant health. The local climatic conditions (Table 1) during our study also were favorable for corn production, as the state of Mississippi set record corn yields in 3 of the 4 years, and experienced an overall increase of 36% compared to the previous 10-year average (NASS). These conditions were not favorable for mycotoxin contamination and we did not detect any CPA or fumonisin during this experiment. Aflatoxin was only detected in 20 of the 88 kernel samples in 2012, and only three of the 88 kernel samples exhibited AF concentrations greater than 5 ppb. In 2013, there were 13 aflatoxin-positive samples, with nine having concentrations greater than 5 ppb. Six positive samples were found in 2014, five of which were over 5 ppb. In 2015, the final year of the study, no aflatoxin was found in any of the samples. There was no significant effect of treatment on the aflatoxin concentration in the harvested corn.

### Colonization of Soil by *A. flavus*

**Figure 2:**
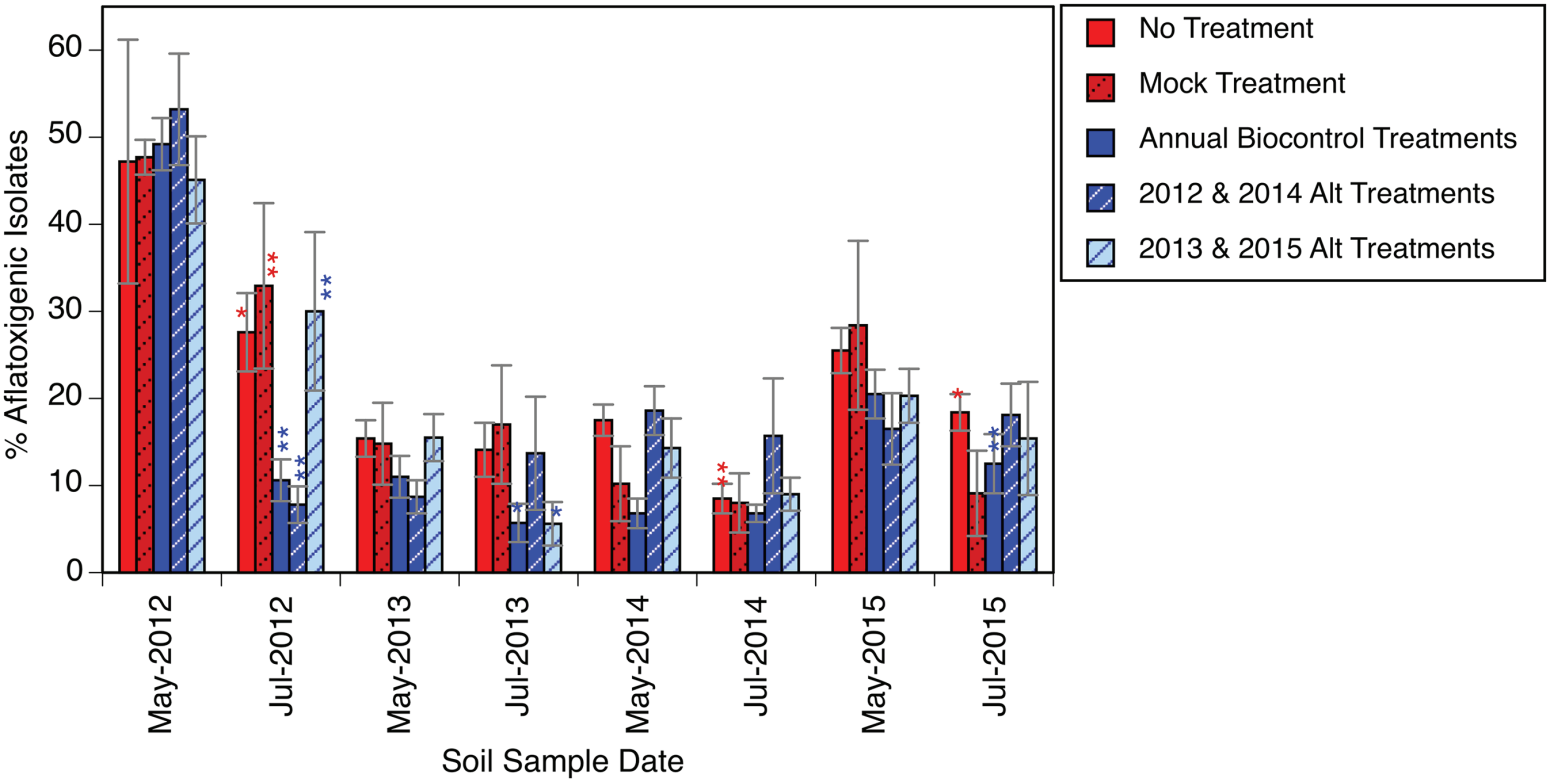
Relative abundance of presumptive aflatoxigenic isolates in soil over time in response to biocontrol applications. Asterisk or asterisks used to indicate a significant (*α* = 0.05) or highly significant (*α* < 0.001) reduction, respectively in the percentage of presumptive aflatoxigenic isolates between a May and July sampling date within plots of a given treatment. Error bars indicate one standard error of the mean.

The percentage of aflatoxigenic isolates collected from the soil is presented in Figures 1B–F, 2. At the initiation of the experiment (Figure 1B), and before any biocontrol treatments were applied, the soil *A. flavus* population was determined to be about 48% (standard deviation = 15%) aflatoxigenic. Approximately 2 months after the first phase of biocontrol products were distributed in their respective plots, each treatment area had a significantly lower frequency of aflatoxigenic isolates (Figure 1C). The plots that were to receive four annual biocontrol treatments went from harboring 49% aflatoxigenic *A. flavus* to 11% aflatoxigenic *A. flavus,* and the plots to receive biennial treatments (2012 and 2014) went from 53% aflatoxigenic *A. flavus* to just 8%. This reduction was to be expected, and is the purpose of the biocontrol application; however the co-occurrence of significant reductions in untreated plots that did not receive biocontrol treatments was not as obvious. Others have reported detection of biocontrol strains outside of the treatment area (Cotty, 1994; Bock et al., 2004). In the present study, we not only detected atoxigenic strains in untreated areas, but we observed a dramatic shift in the population in these untreated areas. Many of the “untreated” plots, scheduled to receive biocontrol treatment in subsequent years (2013 and 2015), shared a border with treated plots and may have benefited from a halo effect from an adjacent treatment.

The mock inoculated treatment, which only received an application of sterilized wheat, also had a highly significant aflatoxigenicity shift: between May and July, the *A. flavus* population in plots that received no biocontrol treatment shifted from 48% aflatoxigenic to about 30% aflatoxigenic. By the following May, less than 20% of the strains isolated from these treatment areas were aflatoxigenic. Plots in these treatments were separated from plots with biocontrol treatments by a 20-m buffer, as indicated in Figure 1A. It is possible that the passive spread of these biocontrol strains, by normal agricultural field work, windborne spore dispersal, drift during occasional flooded conditions, or movement by insects or animals influenced the conditions of this experiment.

Plot-by-plot examination of the soil population over time reveals other patterns. In July 2012 (Figure 1C), nearly all of the plots with >20% aflatoxigenic isolates were either untreated buffers (plots with black outlines) or plots that would not receive their first treatments until the following year (plots with yellow outlines). In 2013 and 2014 (Figures 1D–G, respectively), many of the plots with >20% aflatoxigenic isolates were along the margins of the field; the North and South margins in 2013 and the South and East margins in 2014. If there was substantial movement of inoculum from the biocontrol treatments into nearby plots, it might be expected that plots on the edge would not shift as quickly as the central portion of the field. Results from 2015 are indicative of a shift in the population to a slightly more aflatoxigenic state, but still much less toxigenic than the initial, 2012 conditions.

The five treatments were compared for aflatoxigenicity shifts between May and July of each of the 4 years, for 20 comparisons in total (Figure 2). In the first year, as discussed above, all five treatments had a statistically significant shift toward a less toxigenic state. In the following 3 years, only five more occurrences of significant reductions in aflatoxigenic percentage were detected. Thus, of all the instances where the *A. flavus* population had a statistically significant shift, half of them were in the first year of treatments. The 10 occurrences of significant changes in July were also associated with comparatively highly aflatoxigenic states in May; i.e., measurable reductions in the aflatoxigenic percentage were more common when the starting population was relatively highly aflatoxigenic. Across all treatments, over the course of the entire experiment, the soil *A. flavus* population was 33% (S.E. = 5%) aflatoxigenic in May for the treatments that would have a significant reduction in the aflatoxigenic percentage by July, and just 15% (S.E. = 2%) in treatments that would not have a significant reduction. A reasonable inference is that the expense of a biocontrol product application is most likely to be warranted when the initial population is highly toxigenic, and that attempting to further shift an already less toxigenic population is not as likely to be effective. It is possible that it is simply difficult to detect a meaningful shift to a lower percentage when starting from a fairly low percentage. It is also reasonable for the biocontrol products to have limited efficacy when the background population has minimal aflatoxigenicity.

### Colonization of Grain by *A. flavus*

**Figure 3:**
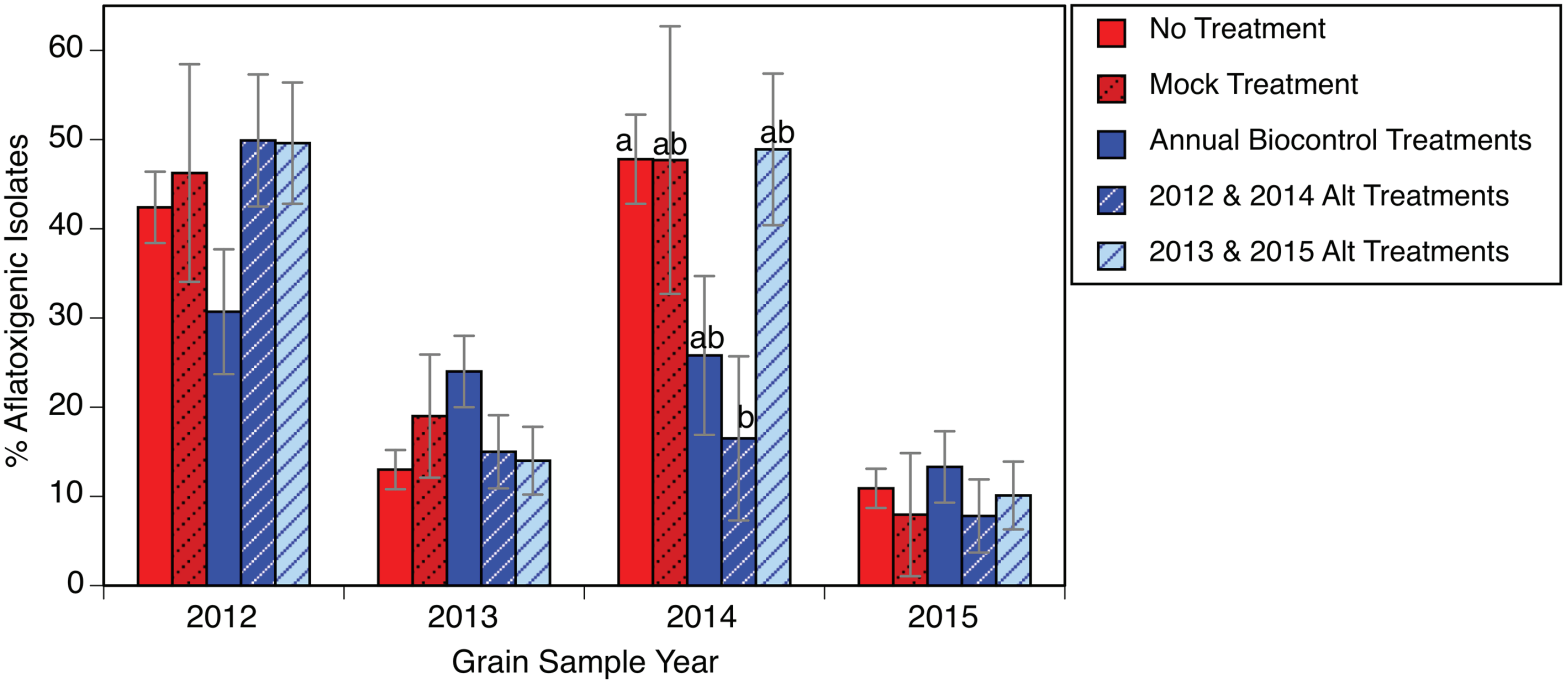
Relative abundance of presumptive aflatoxigenic isolates in grain samples in response to biocontrol applications. Bars within the same year with different letters are significantly different by Tukey’s honestly significant difference test. Error bars indicate one standard error of the mean.

In 3 of the 4 years encompassed in our study, it was not possible to discern a significant effect from the biocontrol treatments on the *A. flavus* populations that colonized the grain (Figure 3). Only in 2014 did one of the treatments, a biennial biocontrol application, have an *A. flavus* population with a significantly lower percentage of aflatoxigenic isolates. This “failure” of the biocontrol, however, must be seen in context. Even if the biocontrol applications did not significantly shift the population on the grain to a less aflatoxigenic state at the time of harvest, there was still nearly zero aflatoxin observed on the corn samples. Also, in two of the years without significant effect, the background *A. flavus* population (the untreated or mock inoculated plots) was only 10–20% aflatoxigenic, making it impossible for us to detect any further reduction with the resolution of our analytical methods. Furthermore, this experiment did not include any of the stresses commonly associated with high aflatoxin occurrence such as extremely high temperatures, drought stress, or abundant activity by ear-feeding insects, so the full value of the biocontrol applications may be somewhat masked. The reason for our observed overall increase in aflatoxigenic isolates from 2013 to 2014 is unknown and warrants further investigation. It has been suggested from vegetative compatibility group lineages that the *A. flavus* community has great variability from year to year, even within a given field (Bandyopadhyay et al., 2016). The frequency of aflatoxigenic isolates collected from all corn samples declined from a high of 43% (S.E. = 2.8%) in 2012 to 11% (S.E. = 1.5%) in 2015, consistent with an overall shift in the field population to a minimally aflatoxigenic level.

The experiment intended to address three hypotheses. Within the parameters of the current study, the *A. flavus* population in the soil shifted from 40 to 50% aflatoxigenic to consistently below 20% aflatoxigenic (hypothesis 1, Figure 2). In the final year of the experiment, there were several plots that appeared to revert from the “<20% aflatoxigenic” to the “20–40% aflatoxigenic” status (Figure 1). This could reflect weather conditions that year, migration of a different population, or allelic drift. Even with this change in the final year, the percentage of aflatoxigenc isolates was still less than half of the starting field average. The *A. flavus* population colonizing the grain from this field also declined, ending at approximately 10% aflatoxigenic (Figure 3). There was no evidence in the present experiment to support the expense of repeated, annual applications of biocontrol products. Similar reductions in toxigenic *A. flavus* were observed in plots that received biocontrol applications in alternating years (hypothesis 2). The shift to a lower frequency of aflatoxigenic isolates in untreated plots is consistent with the hypothesis that there is significant movement of inoculum (hypothesis 3). This observation could support alternative, more economical application methods.

## Conclusion

While aflatoxin was nearly undetectable in the present experiment, it was possible to quantify the *A. flavus* population in soil and grain samples to monitor the decrease in the relative abundance of aflatoxigenic isolates of *A. flavus*. In the soil, significant aflatoxigenicity shifts were most common in the first year of biocontrol application. Biocontrol efficacy was positively associated with a relatively high frequency of aflatoxigenic isolates in May, immediately before application, but generally did not produce a significant effect if the background population was already <15% aflatoxigenic. Significant population changes in untreated plots were consistent with substantial passive movement of *A. flavus* inoculum beyond 20 meters. After 4 years of biocontrol applications, the *A. flavus* population recovered from the grain was approximately 11% aflatoxigenic, regardless of the particular biocontrol treatment. Biocontrol applications are most likely to be beneficial when the initial soil population has a high percentage of aflatoxigenic isolates. Future studies should be initiated when aflatoxin levels are at greater concentrations, and also should include a single biocontrol application treatment at year one, with no other applications in subsequent years, to determine if the number of aflatoxigenic strains increases to pre-application levels.

## Data Availability

The datasets generated for this study are available on request to the corresponding author.

## Author Contributions

MW planned and executed the experiment, analyzed results, and wrote the manuscript. HA participated in planning the experiment, provided technical support to sample collection, and reviewed the manuscript.

### Conflict of Interest Statement

The authors declare that the research was conducted in the absence of any commercial or financial relationships that could be construed as a potential conflict of interest.
